# When scoliosis surgery in a child unexpectedly resolves hydronephrosis: A case report and review of the literature

**DOI:** 10.1016/j.eucr.2025.103138

**Published:** 2025-07-21

**Authors:** Peranzoni Francesca, Zambelli Pierre-Yves, Broennimann Enrico

**Affiliations:** Department of Pediatric Surgery, University Hospital of Lausanne (CHUV), University Center of Pediatric Surgery of Western Switzerland, Lausanne, Switzerland

## Abstract

Hydronephrosis, defined as dilation of the renal pelvis and calyces, may occur with scoliosis or after corrective surgery. However, spontaneous resolution of pre-existing hydronephrosis following scoliosis correction has not been previously reported. We present a 12-year-old girl with a *ZMYM2* mutation, severe thoracolumbar scoliosis (Cobb 103°), and bilateral hydronephrosis. After posterior spinal fusion (T4–L3), hydronephrosis resolved completely. Just as the spinal angle, likely the angle between the renal pelvis and ureter was similarly adjusted, facilitating improved urinary drainage. This rare case highlights a possible dual therapeutic effect of scoliosis correction on spinal alignment and urinary drainage in selected cases.

## 1 Introduction

Congenital scoliosis (CS) and hydronephrosis are pathological conditions that can significantly impact patients' quality of life.

CS is a form of spinal curvature caused by underlying congenital vertebral malformations, clinically defined as a lateral curvature of the spine exceeding 10° with concordant vertebral rotation.^1^ Concomitant congenital deformities are common, particularly in the genitourinary, respiratory, and cardiovascular systems, which share a common embryological origin from the mesoderm.^2^

Hydronephrosis is defined as an abnormally large pelvicalyceal system,^3^ and it is occasionally observed in association with scoliosis, most likely in patients with mean Cobb angles exceeding 50°.^4^ This relationship extends beyond the initial presentation, as hydronephrosis has been noted as a potential complication following scoliosis corrective surgery. Interestingly, while post-surgical hydronephrosis is documented, the medical literature lacks reports of pre-existing hydronephrosis resolving after scoliosis correction.^5^^,^^6^

This report presents a unique case of a 12-year-old girl with a ZMYM2 mutation, concomitant scoliosis, and bilateral hydronephrosis. Remarkably, the patient's hydronephrosis entirely resolved following surgical scoliosis correction.

## 2 Case report

**Fig. 1:**
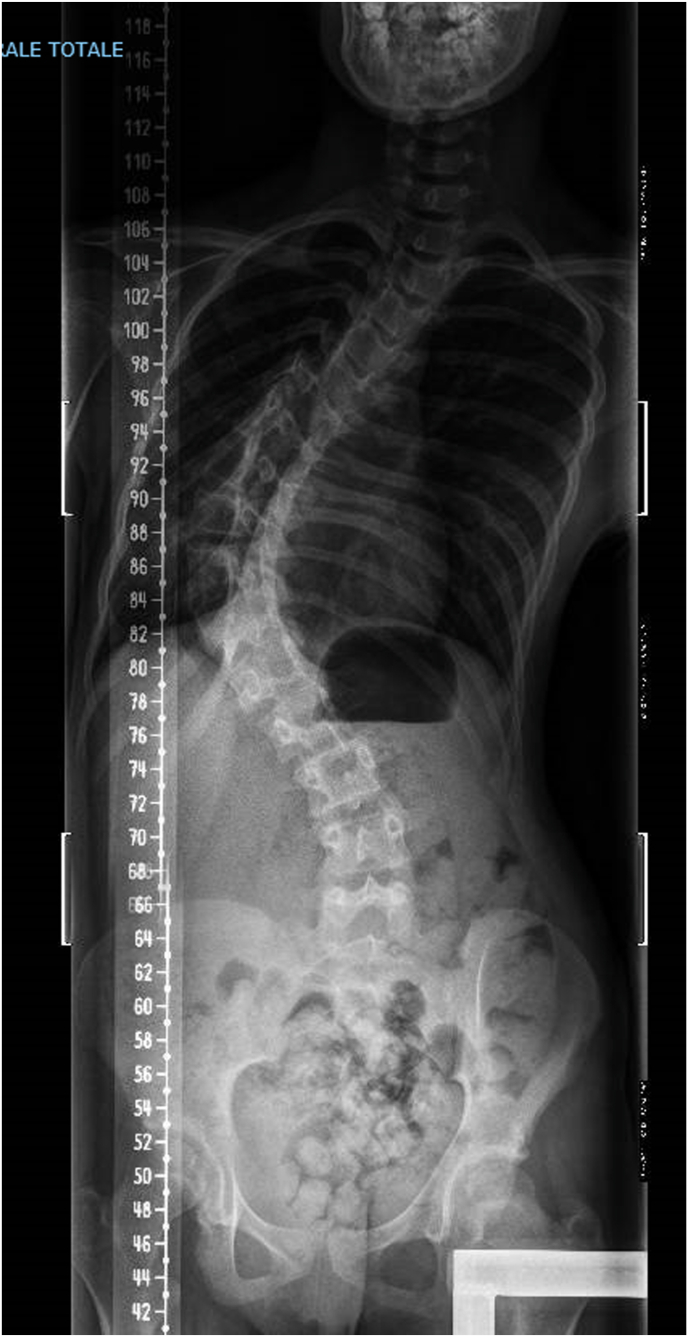
Severe scoliosis of the patient at 12 years of age.

Our young patient has a developmental disorder (affecting cognitive-behavioural, language development, learning, and motor development), autism spectrum disorder, and Ehlers-Danlos syndrome with significant hyperlaxity and severe juvenile dextrorotatory scoliosis (Fig. 1).

**Fig. 2:**
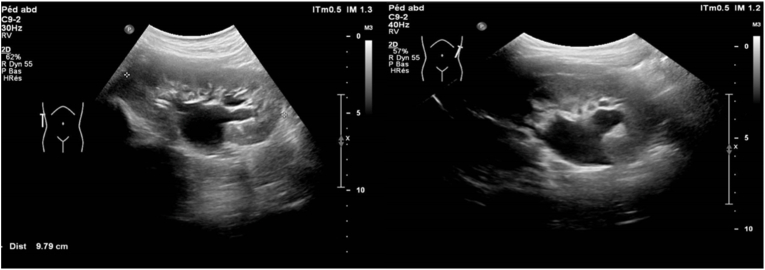
Right pelvis measuring 16 mm and left pelvis measuring 13 mm.

Genetic analysis performed at 10 years of age revealed heterozygosity of the ZMYM2 gene (variant c.2140C > T, p.Gln714∗), corresponding to a neurodevelopmental-craniofacial syndrome with variable renal and cardiac abnormalities. Ultrasound screening at the age of 10 years for urinary tract malformation showed bilateral hydronephrosis classified as urinary tract dilation P2, with suspicion of lower polar arteries on both sides. There was bilateral central and peripheral calyceal dilation, with the pelvis measuring 16 mm on the right and 13 mm on the left (Fig. 2). No other imaging modalities were performed as contrast-enhanced cross-sectional imaging or preoperative renal scintigraphy.

The distal ureters were not dilated, and the bladder appeared unremarkable

According to the patient's medical history, she is dry during the day and night and has regular urination. She experienced a single episode of cystitis at age 6. During the clinical examination by the pediatric urologist, she did not exhibit significant flank pain.

Echocardiography screening showed no abnormalities

**Fig. 3:**
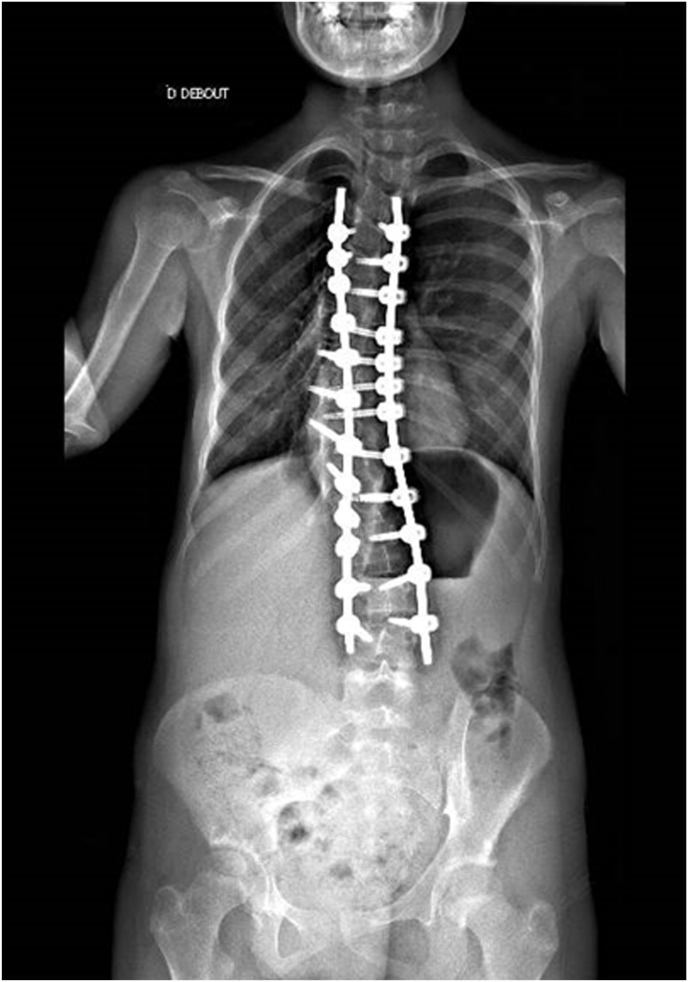
Images following surgical correction of scoliosis.

Regarding the scoliosis, at 5 years old, the patient was prescribed a corset due to the diagnosis of asymptomatic dextro-convex thoraco-lumbar scoliosis, measured at 56° according to Cobb, centered on T6-L1. At age 11, considering the persistence of scoliosis with a Cobb angle between T7-L1 of 103° (Fig. 1) and related symptoms such as extreme spine rigidity and cutaneous complications from constant corset use, surgical correction was deemed necessary. At 11 years of age, the patient underwent scoliosis correction by T4-L3 arthrodesis via posterior approach after HALO traction (max 12kg traction) for 6 weeks. The intraoperative and 3-month postoperative results are satisfactory (Fig. 3).

**Fig. 4:**
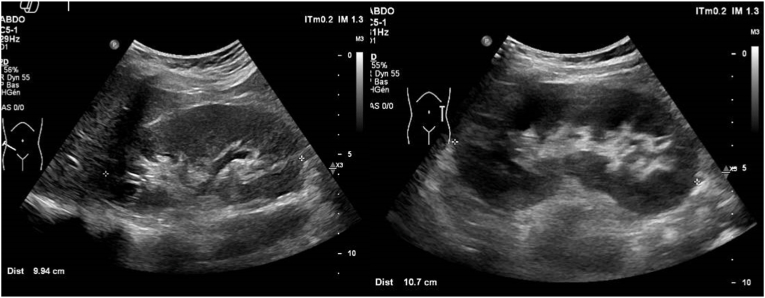
Resolution of bilateral hydronephrosis post-scoliosis correction.

Urological follow-up was performed 3 months and one year after the scoliosis surgery. Ultrasound imaging showed the complete resolution of hydronephrosis on both sides (Fig. 4).

## 3 Discussion

This case report highlights an unusual correlation between scoliosis correction and hydronephrosis resolution in a pediatric patient.

### 3.1 Hydronephrosis

Hydronephrosis can be classified as either obstructed or non-obstructed, depending on the underlying cause. Obstructed hydronephrosis occurs when there's a physical blockage preventing urine from flowing normally, while non-obstructed hydronephrosis may result from other factors such as vesicoureteral reflux.^3^^,^^7^

Mechanical obstructions can be either intrinsic (within the urinary tract) or extrinsic (from outside pressure). These blockages may occur at different points along the urinary tract, including the ureteropelvic junction, ureter, or ureterovesical junction. These obstructions can be caused by factors such as stones, strictures, kinking of the ureter, or external compression^8^

It is important to emphasize that, in the absence of contrast-enhanced cross-sectional imaging or preoperative renal scintigraphy, the precise etiology of hydronephrosis in our patient could not be definitively established. However, potential contributing factors include kinking of the ureter, an abnormal and obstructive angle between the renal pelvis and the ureter, the presence of polar vessels, or extrinsic compression, —any of which may have been influenced by the altered anatomical relationships due to the severe spinal curvature.

### 3.2 Hydronephrosis and renal function

Urinary flow impediment results in elevated hydrostatic pressure within the collecting system. This pressure increase subsequently affects the intraglomerular environment, potentially impacting the glomerular filtration process. The extent of kidney function impairment is directly related to how long the obstruction persists and its severity. Failure to alleviate the blockage may result in renal scarring and irreversible damage, compromising both glomerular and tubular functions.^9^

The prolonged obstruction causes a dilated collecting system, compression of papillae, and thinning of parenchyma leading ultimately to cortical atrophy and tubulointerstitial fibrosis.^10^ Impairment of sodium reabsorption, disruption of urinary acidification leading to metabolic acidosis, and urinary concentrating ability are some of the physiologic effects.^11^

### 3.3 Hydronephrosis and scoliosis

The literature emphasizes the relationship between lumbar scoliosis and the concomitant presence of hydronephrosis. This relationship seems to be due the anatomical positioning of the kidneys in patients with scoliosis, especially in those presenting with severe degree of curvature.^4^^,^^12^

In severe cases of scoliosis, the spinal curvature modifies the retroperitoneal space, potentially encapsulating the kidney within the lateral edges of the spinal defect. This confinement not only increases the risk of injury from rapid deceleration but also alters the normal anatomical relationships of retroperitoneal structures.^13^

The presence of hydronephrosis is also described as a complication following surgical correction of scoliosis, often due to extrinsic compression on the urinary tract made by scar tissue or hematomas.^5^^,^^6^

Such anatomical changes related with severe scoliosis can have far-reaching effects on renal positioning, vascular relations, and urinary drainage, underscoring the complex interplay between spinal deformities and renal health.^14^^,^^15^

Although no cross-sectional imaging or renal scintigraphy was performed to clarify the underlying mechanism, resolution of hydronephrosis following spinal realignment suggests that anatomical changes facilitated improved drainage from the renal pelvis into the ureter—just as the spinal angle was corrected, it is likely that the angle between the pelvis and ureter was similarly adjusted.

Notably, this is the first reported case showing the resolution of hydronephrosis after surgical intervention to reduce spinal curvature. It highlights the potential for scoliosis correction to resolve associated urological issues, offering new insights into the management of patients with concurrent spinal and urinary tract abnormalities.

## 4 Conclusion

This rare case underscores the intricate interplay between spinal deformities and hydronephrosis, highlighting the potential impact of scoliosis and its surgical correction on renal function and urinary dynamics.

According to the literature, scoliosis, but notably, also its treatment, can lead to the development of hydronephrosis by altering retroperitoneal anatomy and potentially exerting compressive effects on the urinary tract. However, our case demonstrates that treating scoliosis can also have a positive effect, alleviating pre-existing hydronephrosis.

The precise pathophysiological mechanisms underlying these interactions remain incompletely understood, as do the predictive factors determining whether scoliosis correction benefits the urinary system.

The case report in question raises an intriguing possibility: for patients with hydronephrosis, could scoliosis surgery be considered as a potential solution to address two issues simultaneously - the spinal deformity and the renal dilation due to possible anatomical variations?

Further research is needed to understand these mechanisms better and refine patient selection criteria.

## CRediT authorship contribution statement

**Peranzoni Francesca:** Writing – review & editing, Writing – original draft. **Zambelli Pierre-Yves:** Validation, Investigation, Data curation, Conceptualization. **Broennimann Enrico:** Writing – review & editing, Validation, Supervision, Project administration, Methodology, Investigation, Formal analysis, Data curation, Conceptualization.

## Authors statements

Authors declare no conflict of interest.

## Funding

No funding was needed to realise this report.
